# Risk of autoimmunity, cancer seeding, and adverse events in human trials of whole-tissue autologous therapeutic vaccines

**DOI:** 10.1016/j.cpt.2024.05.003

**Published:** 2024-05-31

**Authors:** Garrett Gianneschi, Anthony Scolpino, James Oleske

**Affiliations:** a Department of Neurology, Rutgers University - New Jersey Medical School, Newark, NJ 07103, USA; b Division of Immunology, Department of Pediatrics, Rutgers University - New Jersey Medical School, Newark, NJ 07103, USA

**Keywords:** Neoplasms, Vaccination, Autoimmunity, Immunotherapy, Drug-related side effects and adverse reactions, Systematic review, Cancer vaccines

## Abstract

**Background:**

Whole-tissue autologous therapeutic vaccines (WATVs) are a form of cancer immunotherapy that use a patient's own pathological tissue. Concerns exist regarding the potential of WATVs to induce autoimmunity or the spread of cancer; however, their adverse events (AEs) have not been adequately studied. This literature review primarily aimed to evaluate the risks of autoimmunity and cancer seeding associated with using WATVs in human clinical trials. Its secondary objectives included assessing the incidence of AEs graded 1–5 using the Common Terminology Criteria for Adverse Events v5.0.

**Methods:**

The inclusion criteria were any clinical trial using human subjects in which at least part of the cancer vaccine was derived from the patient's own tumor tissue, which likely preserved the unique tumor-associated antigens (TAAs) present in the patient's tumor (i.e., whole-tissue). Tumor vaccine trials that used limited TAAs or highly processed tumor antigens were excluded. Published clinical trials were searched using Google Scholar until March 2024. The authors elaborated on the risk of bias in such cases, as indicated. All reviewed publications were searched for evidence of autoimmunity, cancer seeding, and other AEs. The Preferred Reporting Items for Systematic Reviews and Meta-Analyses 2020 statement guided the review.

**Results:**

Data from 55 human clinical trials, abstracts, case reports, and unpublished data were analyzed, including 3323 patients treated with WATVs for various cancers. The primary outcomes were: (1) no documented cases of WATV-induced autoimmunity, (2) no documented cases of WATV-induced spreading or seeding of noninfectious cancers, and (3) the observed 0.24% (2/838) risk of spreading or seeding infectious cancers was attributed to inadequate sterilization. The secondary outcomes were: (1) no deaths were attributed to WATV therapy, (2) 0.18% (6/3323) incidence of grade 4 AEs, (3) 0.42% (14/3323) incidence of grade 3 AEs, (4) the incidence of grades 1–2 AEs was 52.21% (478/916).

**Conclusions:**

WATVs carry no risk of inducing autoimmunity and essentially no risk of cancer seeding if properly sterilized. WATVs also exhibit a side effect profile comparable to that of routine vaccinations, with common, mild, and transient adverse effects. The combined risk of grade 3 and 4 AEs was 0.60% (20/3323). No deaths were causally associated with WATV treatment.

## Highlights

• Fifty-five cancer trials using whole-tissue autologous therapeutic vaccines (WATV) were included in this systematic review.
• There was no incidence of death or autoimmunity across 3323 pooled patients.
• There was a 0.24% (2/838) risk of seeding in infectious cancers and no risk of seeding in noninfectious cancers.
• WATV use poses risks for grades 3 and 4 adverse events (AEs) at 0.42% (14/3323) and 0.18% (6/3323), respectively.
• WATV use poses a risk for grades 1–2 AEs at 52.21% (478/916), similar to inactivated vaccines.

## Graphical abstract

**Figure:**
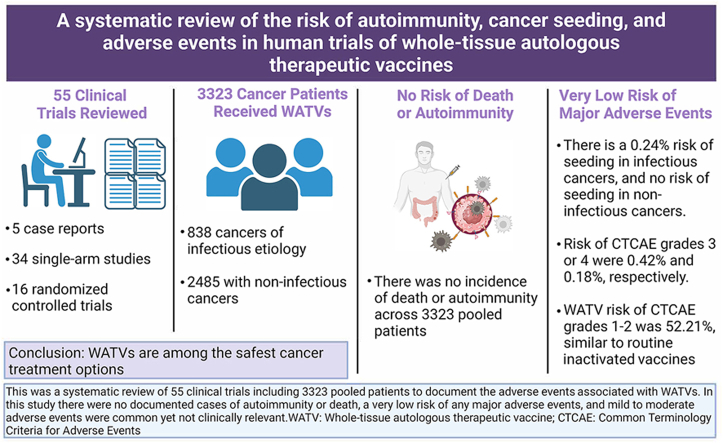

## Introduction

Whole-tissue autologous therapeutic vaccines (WATVs) are a personalized form of cancer immunotherapy that harnesses a patient's own tumor tissue to stimulate an immune response against a disease. Despite their long history of safe and promising use against both cancers and infectious diseases, WATVs remain an underutilized and understudied treatment option. One way to increase their popularity is to better understand their safety profile, which was investigated in this study.

Concerns exist regarding the potential of WATVs to induce autoimmunity or promote the spread of cancer; however, no systematic review has comprehensively addressed these risks. Similarly, systematic analyses of the general adverse events (AEs) of WATVs are lacking. While a similar meta-analysis was proposed by Khan et al.^1^ in 2020, their results remain unpublished. The adjuvants and combination chemotherapies used in WATV therapy have more documented AEs than the WATVs themselves. Therefore, this review has two objectives. First, it aimed to assess the risk of WATV-induced autoimmunity or cancer seeding. Second, we evaluated the risk of WATV-induced AEs using the Common Terminology Criteria for Adverse Events (CTCAE) scale. This review demonstrated that WATVs are associated with minimal AEs. Their favorable safety profile, combined with their potential for personalization, makes them attractive and important areas of cancer research.

The use of WATVs dates back to Biberstein's^2^ success with wart and condyloma treatments in 1925. After that, WATVs became a popular method for treating cancers of infectious etiology. This early success in treating cancers of infectious etiology then spilled over to noninfectious cancers. Many early attempts have been made to identify cancers that respond best to WATV therapy. The production methods of WATVs from the 1920s through the 1970s mostly relied on crude mechanical disruption, heat, and chemical sterilization of the tumor tissue. The most popular adjuvants then were Freund's adjuvant, Bacillus Calmette-Guérin (BCG), and WATVs alone. Modern production methods use various cell lysis techniques or the chemical separation of individual tumor cells. Newer adjuvants include cancer chemotherapies or WATVs genetically modified with granulocyte-macrophage colony-stimulating factors (GM-CSFs). Between the late 2000s and the mid-2010s, WATV research declined as cancer treatment shifted toward targeted therapies, such as those focused on allogeneic vaccines, specific tumor marker antibodies, dendritic cell vaccines, and chimeric antigen receptor T-cell (CAR-T) therapy. However, renewed interest in WATVs has recently developed, as evidenced by large clinical trials, such as OncoVAX, Vigil, and Reniale vaccines, which are actively being studied for colon, breast/ovarian, and renal cell carcinoma (RCC), respectively. A large recent study by May et al.^3^ investigated the use of Reniale in 692 patients with RCC. This successful trial showed significantly improved overall survival rates in the Reniale group compared to the control group, particularly for patients with advanced-stage RCC.^3^ Vigil has been the most actively researched WATV in recent years and has been included in two separate trials within the past 2 years.^4^^,^^5^ None of these therapies have become Food and Drug Administration (FDA)-approved; however, Vigil has been granted Fast Track designation by the FDA.

WATVs have a mechanism of action similar to conventional vaccines. In particular, conventional vaccines expose the immune system to viral or bacterial antigens, prompting it to learn and develop defenses against specific pathogens. Similarly, in cancer therapy, WATVs utilize tumor-associated antigens (TAAs) isolated from a patient's own tumor tissue. When combined with an adjuvant, TAAs stimulate the immune system to recognize and destroy cancer cells. Reactive leukocytosis is then induced, much like what would be seen in an infection that generates lymphocytic infiltration and tumor tissue destruction.^6^^,^^7^ Inducing the body into a heightened immunologic state is also responsible for the flu-like symptoms commonly seen in infections and WATV-induced AEs. WATVs offer a unique advantage over other cancer therapies by preserving the complete range of TAAs in patients. These TAAs are highly personalized and likely only exist in a specific patient.^8^ This personalized approach ensures that the vaccine targets the exact profile of a tumor. This highly individualized approach creates a powerful customized cancer therapy with the potential to minimize tumor escape mechanisms.

The CTCAE system is a widely accepted and standardized approach for categorizing AEs in cancer treatment. An AE is defined as any unusual medical issue that occurs during cancer treatment, even if the treatment does not directly cause it. The current version of the CTCAE (v5.0) uses grades to describe side effect severity. Grade 0 indicates no AEs; grade 1, mild; grade 2, moderate; grade 3, severe but not immediately life-threatening; grade 4, life-threatening; and grade 5, death.^9^

This systematic review included unpublished observations of a clinical trial conducted by the authors with permission from the authors. The Pediatric Laryngeal Papilloma clinical trial conducted between 1999 and 2011 investigated the use of WATV in 50 children with refractory juvenile papilloma of the larynx (NCT00002454).^10^ There were no severe AEs associated with WATV in this trial, and approximately 90% of cases achieved complete resolution of papillomas within 3–6 months. This technique was first described in a 1971 case report by Oleske and Kushnick.^11^

## Methods

### Information sources and search strategies

**Figure 1:**
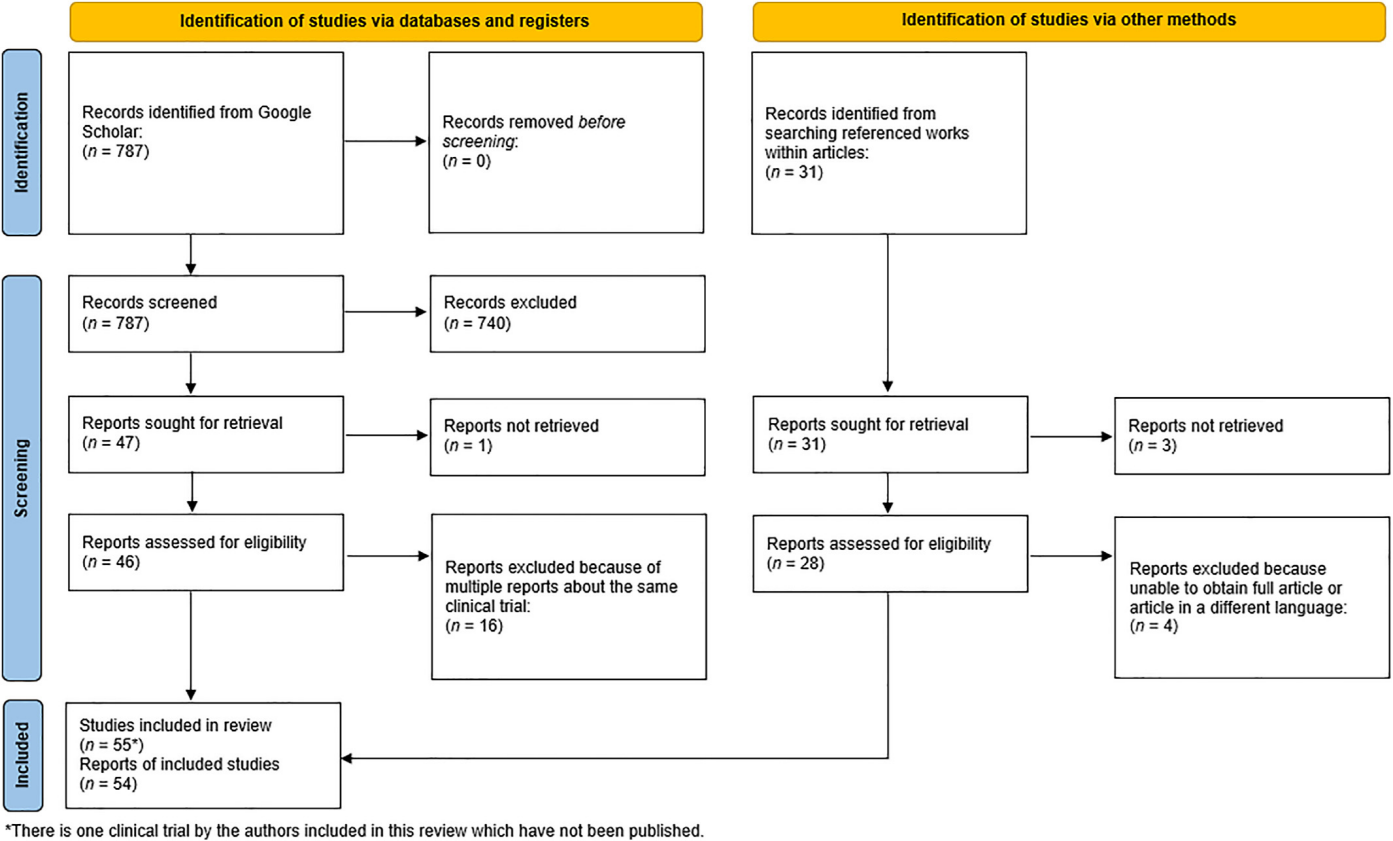
Flow chart of the literature search, screening, and final inclusion.

We conducted a comprehensive search for relevant studies between March 2023 and February 2024 using Google Scholar. The search terms employed were “autologous”, “cancer”, “vaccine”, “clinical trial”, “whole tumor”, and “human”. The initial search yielded 354 results. An additional search was conducted using the terms “whole tumor cell vaccine”, “human”, and “trial”, resulting in 433 additional entries. The search results are displayed in Figure 1.

A two-step screening process was used to identify eligible studies. The titles and abstracts of all retrieved studies (*n* = 787) were initially screened by a single reviewer to exclude irrelevant articles (e.g., non-human studies, studies not related to WATVs, and duplicate studies). This initial screening yielded 47 unique articles and abstracts for further analysis. The references within these 47 articles generated 31 additional unique articles for review purposes. Seven of these 31 articles were not reviewed because they were in a different language or the full article/abstract could not be retrieved.

Following initial screening, full-text articles were retrieved and reviewed by three independent reviewers to ensure that they met the inclusion criteria. Studies with ongoing publications that update the same clinical trial at different time points were reviewed based on the most recent publication available to avoid counting the same patient multiple times. Thirty articles from the initial Google Scholar search and 24 articles identified through reference screening were evaluated. This review also incorporated data from one of our unpublished open-label clinical trials, As previously described.^10^ To minimize reporting bias, all studies were initially screened by one reviewer and subsequently confirmed to meet the review criteria by two additional reviewers. Automated screening tools were not used in this study.

We designed our search terms to distinguish between WATVs and other autologous therapeutic vaccines (ATVs). However, the numerous ATV subtypes and evolving WATV terminology have complicated this process. These aspects led to a low initial yield of relevant articles, with more studies being found through reference searches. Articles not published in English and those in which it was not specifically stated in the Methods section that the entire tissue was in the final vaccine product were excluded. Notably, the Graham and Graham^12^ 1962 study, often cited as an early large-scale WATV trial, could not be obtained from the Surgery, Gynecology, and Obstetrics archives. In the end, 55 clinical trials, abstracts, and case reports were included in this review. These 55 studies were then grouped into infectious or non-infectious cancers, which were further subdivided into organ systems (lungs, kidneys, mixed, etc.). This systematic review followed PRISMA reporting guidelines.

### Inclusion and exclusion criteria

This systematic review included studies that met the following criteria: (1) studies that involved human participants enrolled in a clinical trial, and (2) the administered cancer vaccine had to comprise at least partially the patient's own tumor tissue, preserving the unique TAAs. Trials that utilized a limited number of preselected TAAs or highly processed tumor vaccines (e.g., dendritic cell vaccines loaded with autologous tumor antigens) were excluded.

### Data collection

The authors pooled patient AE data from all 55 included studies to measure the incidence and risk of WATV AEs. When reviewing the studies, it was presumed that all 55 studies recorded and published all the significant medical events of AE grades 3–5, including seeding/spreading and autoimmunity. Furthermore, it was presumed that AE grades 1–2 were not reliably reported unless a specific incidence was reported. We recorded the incidence, type, and severity of all reported AEs, which were then labeled according to their best-fit grade on the CTCAE v5.0. For instance, Vermorken et al.^13^ recorded skin necrosis requiring surgical excision and discontinued treatment in one of 128 patients; therefore, we counted that as a grade 3 AE. Moreover, researchers such as Barve et al.^4^ specifically stated that some AEs were unlikely due to WATV therapy and are more likely due to the accompanying chemotherapy; therefore, they were not recorded in this analysis.

### Risk of bias

The risk of bias in each study was determined by two reviewers who did not work independently. Moreover, the risk of bias was limited to the quality of the adverse effects reported. The inclusion of certain adverse effects in the pooled patient analysis is described in the next section.

### Synthesis methods and statistical analysis

After data collection on the adverse effect types, the incidence, grades, and AEs were pooled for analysis. The incidence of each AE was calculated as the total number of AEs divided by the total number of patients included. For example, to calculate the incidence and risk of grade 3 AEs, the total number of grade 3 AEs was divided by the total cohort size included in the study, as shown in Equation 1. The same approach was used for grade 4 and 5 AEs, autoimmunity, and seeding.

$\frac{Incidence\;of\;any\;grade\;3\;AE\;\left(14\right)}{Total\;patients\;in\;55\;trials\;\left(3323\right)}$

**Equation 1:** Sample calculation for the incidence of grade 3 adverse events (AEs). AE: Adverse event.

Grade 1–2 AEs were treated differently due to potential underreporting, so we excluded studies that did not explicitly mention the incidence of any grade 1–2 AEs or the number of patients who experienced them. This rationale stems from two observations. Some studies may not have viewed grade 1–2 AEs (e.g., injection site reactions) as true AEs, considering their potential signs of treatment response, as seen in the Sloan et al.^14^ 2000 trial. Moreover, the low number of studies reporting grade 1–2 AEs (20 out of 55) suggested potential underreporting in many studies. For example, the absence of any reported AEs in four studies (McCune et al.,^15^ Malison, Morris, and Jones,^16^ Huang et al.,^17^ and Powell, Pollard, and Jinkins^18^) enrolling a combined total of 99 patients was considered improbable.

Therefore, we excluded studies lacking data on grade 1–2 AEs, assuming that the absence of reported events indicated a lack of serious AEs (grade 3 or above). Our analysis of grade 1–2 AEs focused on the relative number of patients experiencing any grade 1–2 AEs rather than documenting the incidence of each particular AE. This was done because grade 1–2 AEs are typically self-resolving, do not require treatment discontinuation, and are, therefore, of little clinical importance. To calculate their incidence and risk, we divided the number of patients experiencing any grade 1–2 AE (*n* = 478) by the total number of pooled patients within the 20 articles reviewed (*N* = 916), as shown in the sample calculation provided in Equation 2 below. Sensitivity and certainty assessments were not conducted.

$\frac{Subjects\;with\;any\;grade\;1-2\;AE\;\left(478\right)}{Total\;patients\;in\;20\;trials\;\left(916\right)}$

**Equation 2:** Sample calculation for the incidence of grade 1–2 adverse events (AEs). AE: Adverse event.

Many previous WATV studies predated the CTCAE system. To maximize data inclusion, we translated the reported AEs into approximate CTCAE grades. This is generally straightforward based on the descriptions provided in each text. However, some studies reported a range of AE grades (e.g., the Mahvi et al.^19^ study that reported patients to have grade 2 *or* 3 AEs). In such cases, we evenly split the AEs between the two grades for analysis.

## Results

### Search results

This literature review and pooled patient analysis included 55 studies (54 published and one unpublished). Our analysis included 3323 patients, 838 of which had cancers of infectious etiology and 2485 had non-infectious cancers. WATVs were investigated in cancers of various organ systems. The most well-studied cancer is RCC at 1296 patients, cancers of infectious etiology at 838 patients, and colon cancer (CC) at 444 patients. This review included five case reports, 34 single-arm studies (SASs), and 16 randomized controlled trials (RCTs). All included studies spanned nearly a century, from 1925 to 2022. Information about these studies are included in Supplementary Table 1, including the authors, year the article was published, disease or organ systems involved, documentation of grade 1–2 AEs, documentation of grade 3–5 AEs, adjuvants, and other components added to the WATV, and a brief description of the study design and results.

### Incidence of autoimmunity and seeding

The primary purpose of this study was to address theoretical concerns regarding autoimmunity and seeding in WATVs, as summarized in Table 1. No cases of WATV-induced autoimmune events were reported across the pooled patients (*N* = 3323). Similarly, no autoimmune events attributed to the WATV AEs were reported in any of the 55 reviewed studies. All studies were included in the assessment of the risk of autoimmunity in WATVs.

**Table 1:** Incidence of WATV-induced autoimmunity and seeding from 3323 patients receiving WATV therapy for cancer.

Autoimmunity and seeding	Incidence, *n*/*N* (%)
Autoimmunity in any neoplasm	0/3323 (0)
Seeding in any neoplasm	2/3323 (0.06)
Seeding in infectious neoplasm	2/838 (0.24)
Seeding in non-infectious neoplasm	0/2371 (0)

WATV: Whole-tissue autologous therapeutic vaccine.

Across all pooled patients, the incidence of tumor seeding following WATV therapy was 0.06% (2/3323). In the subgroup analysis, the patients were divided into the non-infectious cancer group (*n* = 2485) and the cancer of likely infectious etiology (*N* = 838) group. Non-infectious cancer trials reported a 0% incidence rate and never reported seeding events. Cancers of likely infectious etiology had a 0.24% incidence (2/838) of cancer seeding, which is limited to older studies on warts. Biberstein^20^ and Cormia^21^ noted wart development at the injection site in these two cases, which Biberstein attributed to incomplete heat inactivation of the virus during tumor vaccine preparation. Of note, cancer seeding has not been reported in the past 80 years of clinical trials.

### Incidence of adverse events

The secondary objective of this review was to understand the risk of WATVs in causing AE grades 1–5. The data are summarized in Table 2. No deaths were attributed to WATV when assessing the 3323 pooled patients in any of the 55 reviewed studies. All studies were included in the assessment of the risk of death due to WATVs.

**Table 2:** Incidence of WATV-induced AEs reported among 3323 pooled patients for grade 3–5 AEs and 916 pooled patients for grade 1–2 AEs.

Severity	Incidence, *n*/*N* (%)
Grades 1–2	478/916 (52.21)
Grade 3	14/3323 (0.42)
Grade 4	6/3323 (0.18)
Grade 5	0/3323 (0.00)

AE: Adverse event; WATV: Whole-tissue autologous therapeutic vaccine.

Grade 4 AEs had an incidence of 0.18% (6/3323) in the pooled patient analysis of all 55 included studies. Nine patients from the study by Nemunaitis et al.^22^ had grade 3 or 4 AEs, which, in this study, were split into five incidences of grade 4 AEs, which included dyspnea, respiratory distress, chest pain, back pain, pulmonary embolus/tachycardia. One patient from the study by Shipkowitz et al.^23^ had a grade 4 AE from fever and atelectasis of the right middle and lower lung lobes, which led to treatment discontinuation. The individual risk of these grade 4 AEs was <0.18%.^23^

The incidence of grade 3 AEs was 0.42% (14/3323) in all included studies. Five of the 55 included studies reported grade 3 AEs attributed to WATVs.^5^^,^^13^^,^^19^^,^^22^^,^^24^ The individual risk of any specific grade 3 AE was <0.42%, including gait disturbance, back pain, headache, dyspnea or respiratory distress, chest pain, pulmonary embolus/tachycardia, transient lymphocytopenia (without total lymphocyte depletion), and skin necrosis. GM–CSF–transfected varieties of WATVs were present in 92.9% (13/14) of grade 3 AEs, with the remainder being attributed to skin necrosis in the WATV + BCG study.^13^

The documentation of grade 1–2 AEs was of poor quality across all studies examined. In particular, 86.5% (49/55) reported grade 1–2 AEs, three specifically reported no AEs, and four failed to mention any AEs. A low-quality pooled patient analysis (*N* = 916) of 20 studies showed an incidence of 52.21% (478/916) for any grade 1–2 AE. Injection site reactions (induration, ulceration, desquamation, granuloma, abscess, pain, rash, erythema, and swelling), lymphadenopathy, fatigue, and mild fever were the most common; headaches, diarrhea, and stomachaches occurred less frequently. All grade 1–2 AEs were self-limiting and generally resolved within days (except indurations, which could persist for weeks to months). There was no evidence of grade 1–2 AE causing treatment discontinuation.

## Discussion

This study aimed to address the commonly held belief that cancer therapy using WATVs poses a risk of autoimmunity and the spreading or seeding of cancer cells. The general interpretation of this study is that WATV therapy is safe and mostly dependent on additives added to WATV itself. Our results show that the current theoretical concerns regarding WATV-induced autoimmunity are unfounded. Similarly, the risk of cancer seeding is overstated because there is no evidence of cancer seeding in non-infectious cancers. In cancers of viral etiology, seeding has not been reported in the past 80 years. In the earliest studies on WATVs, two patients had localized seeding at the vaccine injection site, which was attributed to poor sterilization techniques. Therefore, using modern laboratory practices, seeding is possible but highly improbable.

This study also aimed to address the large gaps in documenting the characteristics, incidence, and severity of WATV-related AEs. When used alone, WATVs appear to have an AE profile similar to that of routine inactivated vaccines, primarily involving injection site reactions and transient flu-like symptoms.^25^^,^^26^ However, adding adjuvants or other chemotherapeutic medications significantly affects the resulting AEs. Newer studies, such as those by Barve^4^ and Roccoci,^5^ and older studies, such as that by Cunningham et al.,^27^ attributed most of the higher-grade AEs to adjuvants. Compared to newer studies, older studies more frequently used highly antigenic adjuvants, such as Freund's adjuvant and BCG, which more often caused higher rates of injection site abscesses and indurations. Using Freund's adjuvant was stopped in the 1970s because of its side effects. In modern studies, injection site reactions tend to be milder and resolve within days rather than weeks. As mentioned earlier, AEs from injection suggest a stronger immune reaction and better prognosis.^14^^,^^28^, ^29^, ^30^, ^31^ Dosage also influences the toxicity profile, and the severity of injection site reactions is directly correlated with the number of cancer cells included in the WATV preparation.^32^ Therefore, AEs are driven by specific adjuvants, combination therapies, and WATV dosage.

The largest limitation of this study was the lack of standardization in AE reporting. This manifests in two ways: (1) the lack of standardization in the *grade* of reported AEs and (2) *underreporting* or the imprecise reporting of grade 1–2 AEs. To overcome the first limitation, we approximated AE severity using the CTCAE system. To overcome the second issue, we excluded more than half of the reviewed studies, considering their contribution to our understanding of the incidence of grade 1–2 AEs. For instance, 87.5% (49/55) of studies mentioned low-grade AEs; however, only 35.7% (20/55) of the included studies could be analyzed for grade 1–2 AEs. Most studies were excluded because their descriptions of grade 1–2 AEs were too vague. However, more recent studies also suffered from a lack of standardized reporting for AEs. For instance, some studies have reported the incidence of patients experiencing an AE, whereas others have reported the incidence of a specific AE among all patients. Since grade 1–2 AEs are of little clinical significance, this study intended to report the incidence of patients suffering any grade 1–2 AEs, rather than tracking the rate of specific grade 1–2 AEs. the former. As more modern WATV clinical trials adopt the CTCAE system and report grade 1–2 AEs more consistently, the specific details regarding grade 1–2 AEs in WATV therapy will be better elucidated.

Another limitation of our review is that many relevant studies were identified through reference searches rather than through the initial search terms used, suggesting that the search criteria we employed were insufficient. However, the number of pooled patients in our analysis was comparable to that proposed by Khan et al.^1^ in their meta-analysis. This suggests that our search methods, although not perfect, were sufficient to capture a sizable cohort.

The final limitation of this study is the differentiation between death attributed to WATVs and death attributed to disease progression. There were no deaths attributed to WATVs observed in this review; however, a possible death from WATV therapy could be missed or falsely attributed to disease progression. However, no specific organ toxicity has consistently emerged from the use of WATVs, and their side effect profile is similar to that of other inactivated vaccines; therefore, it is unlikely that WATV-induced deaths had occurred and were missed.

### Future perspectives

To enhance the effectiveness of WATVs while maintaining safety, future research should focus on three key areas:

1. Minimizing tumor immune evasion: Novel therapies such as CD200AR-L are being investigated to counteract tumor immune evasion mechanisms and suppress the protective tumor microenvironment.
2. Enhancing vaccine immunogenicity: The development of more potent adjuvants, such as ground glass, which is highly immunogenic yet chemically inert, offers a promising approach to improve vaccine efficacy while minimizing adverse effects.
3. Optimizing the patient's immune response: Given the role of immune dysfunction in tumor development, integrating lifestyle modifications with existing medications may provide a sustainable strategy for improving long-term immune function and overall treatment outcomes.

By addressing these aspects, future WATV therapies can potentially achieve a greater therapeutic impact while ensuring patient safety.

## Conclusion

Collectively, our results strongly support the safety of WATVs. We found no evidence of WATV-induced autoimmunity or death, WATV-induced cancer seeding in non-infectious cancers, and an extremely low risk of spreading virally infected cancer cells. While grade 1–2 AEs are common, severe grade 3–4 AEs due to WATV therapy are rare. This suggests that WATVs are among the safest options available for cancer treatment. Moreover, given their favorable safety profile, WATVs should be further developed to maximize their efficacy.

## Authors contribution

Garrett Gianneschi: study concept and design, acquisition of data, analysis and interpretation of data, drafting of the manuscript, and critical revision of the manuscript for intellectual content; Anthony Scolpino: administrative, technical, or material support, and study supervision; James Oleske: administrative, technical, or material support and study supervision. All the authors critically revised and approved the final version of the manuscript.

## Ethics statement

This study was designed in accordance with the ethical guidelines of the *Declaration of Helsinki*.

## Data availability statement

The datasets used in the current study are available from the corresponding author on reasonable request.

## Declaration of generative AI and AI-assisted technologies in the writing process

During the preparation of this work, the authors used Google Gemini Advanced to improve the clarity, readability, organization, and formatting of this manuscript and as a proofreading tool. After using this tool/service, the authors reviewed and edited the content as needed and took full responsibility for the content of this publication.

## Funding

None.

## Conflict of interest

The authors declare that they have no known competing financial interests or personal relationships that could have appeared to influence the work reported in this paper.

## References

[1] Khan S.T., Montroy J., Forbes N. (2020). Safety and efficacy of autologous tumour cell vaccines as a cancer therapeutic to treat solid tumours and haematological malignancies: a meta-analysis protocol for two systematic reviews. BMJ Open.

[2] Biberstein H. (1925). Experiments on immunotherapy of warts and condylomas. Clinical Weekly.

[3] May M., Brookman-May S., Hoschke B. (2010). Ten-year survival analysis for renal carcinoma patients treated with an autologous tumour lysate vaccine in an adjuvant setting. Cancer Immunol Immunother.

[4] Barve M., Aaron P., Manning L. (2022). Pilot study of combination Gemogenovatucel-T (Vigil) and durvalumab in women with relapsed BRCA-wt triple-negative breast or ovarian cancer. Clin Med Insights Oncol.

[5] Rocconi R.P., Stevens E.E., Bottsford-Miller J.N. (2022). Proof of principle study of sequential combination atezolizumab and Vigil in relapsed ovarian cancer. Cancer Gene Ther.

[6] Sobol R.E., Shawler D.L., Carson C. (1999). Interleukin 2 gene therapy of colorectal carcinoma with autologous irradiated tumor cells and genetically engineered fibroblasts: a Phase I study. Clin Cancer Res.

[7] Berd D., Maguire H.C., McCue P., Mastrangelo M.J. (1990). Treatment of metastatic melanoma with an autologous tumor-cell vaccine: clinical and immunologic results in 64 patients. J Clin Oncol.

[8] Sjöblom T., Jones S., Wood L.D. (2006). The consensus coding sequences of human breast and colorectal cancers. Science.

[9] (2017). Common terminology criteria for adverse events (CTCAE) version 5.0.

[10] Oleske J. (1999). NCT00002454.

[11] Oleske J.M., Kushnick T. (1971). Juvenile papilloma of the larynx. Am J Dis Child.

[12] Graham J.B., Graham R.M. (1962). Autogenous vaccine in cancer patients. Surg Gynecol Obstet.

[13] Vermorken J.B., Claessen A.M., van Tinteren H. (1999). Active specific immunotherapy for stage II and stage III human colon cancer: a randomised trial. Lancet.

[14] Sloan A.E., Dansey R., Zamorano L. (2000). Adoptive immunotherapy in patients with recurrent malignant glioma: preliminary results of using autologous whole-tumor vaccine plus granulocyte-macrophage colony-stimulating factor and adoptive transfer of anti-CD3-activated lymphocytes. Neurosurg Focus.

[15] McCune C.S., O'Donnell R.W., Marquis D.M., Sahasrabudhe D.M. (1990). Renal cell carcinoma treated by vaccines for active specific immunotherapy: correlation of survival with skin testing by autologous tumor cells. Cancer Immunol Immunother.

[16] Malison M.D., Morris R., Jones L.W. (1982). Autogenous vaccine therapy for condyloma acuminatum. A double-blind controlled study. Br J Vener Dis.

[17] Huang J., Zhang Z., Huang H., He Y. (1995). Study on solidified tumor vaccine prepared from autogenous cancerous tissue. Chin J Cancer Res.

[18] Powell L.C., Pollard M., Jinkins JL Sr (1970). Treatment of condyloma acuminata by autogenous vaccine. South Med J.

[19] Mahvi D.M., Shi F.S., Yang N.S. (2002). Immunization by particle-mediated transfer of the granulocyte-macrophage colony-stimulating factor gene into autologous tumor cells in melanoma or sarcoma patients: report of a phase I/IB study. Hum Gene Ther.

[20] Biberstein H. (1944). Immunization therapy of warts. Arch Dermatol Syphilol.

[21] Cormia F.E. (1934). Autolysate therapy for verruca vulgaris. Arch Dermatol Syphilol.

[22] Nemunaitis J., Jahan T., Ross H. (2006). Phase 1/2 trial of autologous tumor mixed with an allogeneic GVAX® vaccine in advanced-stage non-small-cell lung cancer. Cancer Gene Ther.

[23] Shipkowitz N.L., Holper J.C., Worland M.C., Holinger P.H. (1967). Evaluation of an autogenous laryngeal papilloma vaccine. Laryngoscope.

[24] Bota D.A., Chung J., Dandekar M. (2018). Phase II study of ERC1671 plus bevacizumab versus bevacizumab plus placebo in recurrent glioblastoma: interim results and correlations with CD4^+^ T-lymphocyte counts. CNS Oncol.

[25] Alfawaz T.S., Alshehri M., Alshahrani D. (2015). BCG related complications: a single center, prospective observational study. Int J Pediatr Adolesc Med.

[26] Song B.J., Katial R.K. (2004). Update on side effects from common vaccines. Curr Allergy Asthma Rep.

[27] Cunningham T.J., Olson K.B., Laffin R., Horton J., Sullivan J. (1969). Treatment of advanced cancer with active immunization. Cancer.

[28] Peng B.G., Liu S.Q., Kuang M. (2002). Autologous fixed tumor vaccine: a formulation with cytokine-microparticles for protective immunity against recurrence of human hepatocellular carcinoma. Jpn J Cancer Res.

[29] Peng B.G., Liang L.J., He Q. (2005). Tumor vaccine against recurrence of hepatocellular carcinoma. World J Gastroenterol.

[30] Peng B., Liang L., Chen Z. (2006). Autologous tumor vaccine lowering postsurgical recurrent rate of hepatocellular carcinoma. Hepato-Gastroenterology.

[31] Ishikawa E., Tsuboi K., Yamamoto T. (2007). Clinical trial of autologous formalin-fixed tumor vaccine for glioblastoma multiforme patients. Cancer Sci.

[32] Simons J.W., Jaffee E.M., Weber C.E. (1997). Bioactivity of autologous irradiated renal cell carcinoma vaccines generated by ex vivo granulocyte-macrophage colony-stimulating factor gene transfer. Cancer Res.

