# Supplementary material for: Risk of autoimmunity, cancer seeding, and adverse events in human trials of whole-tissue autologous therapeutic vaccines
Source: Cancer Pathog Ther. 2024 May 31;3(2):129–34. doi: 10.1016/j.cpt.2024.05.003 (PMC11963168; doi:10.1016/j.cpt.2024.05.003)
Supplement: Multimedia component 1 [file mmc1.docx]

**Supplementary Table 1: Characteristics and overview of the included WATV clinical trials.**

| Study | Year | Disease/organ system | Number | Grades  1–2 | Grades  3–5 | Adjuvant, components | Description and brief results |
| --- | --- | --- | --- | --- | --- | --- | --- |
| Oleske and Kushnick^[1]^ | 1971 | Laryngeal papilloma | 1 | Yes | No | Ground glass | Case report; CR |
| Oleske^[2]^ | 2000 | Laryngeal papilloma | 50 | Yes | No | Ground glass | SAS; unpublished; CR in 80.0% of cases |
| Shipkowitz *et al*^[3]^ | 1967 | Laryngeal papilloma | 51 | Yes | Yes | WATV alone | SAS; 54.9% had a favorable response, unchanged in 25.5%, progressive 5.9%, unevaluable 13.7% |
| Stephens *et al*^[4]^ | 1979 | Laryngeal papilloma | 17 | No | No | WATV alone | SAS; 52.9% resolved, 29.4% favorable, 17.6% no change |
| Powell, Pollard, and Jinkins^5]^ | 1970 | Condyloma acuminata | 24 | UNR | No | WATV alone | SAS; 83.3% CR, 12.5% PR, 4.2% no response. |
| Biberstein^[6]^ | 1944 | Warts and condylomas | 449 | Yes | No | WATV alone | SAS; two different trials showed 75% and 65.4% favorable responses |
| Abcarian and Sharon^[7]^ | 1982 | Condyloma acuminata | 200 | No | No | WATV alone | SAS; 84.0% CR, 11.0% had one remaining lesion, 5.0% had no response |
| Malison, Morris, and Jones^[8]^ | 1982 | Condyloma acuminata | 34 | No | No | WATV alone | RCT; 55.6% CR in the WATV group compared to placebo, 25.0% complete response; *P* = 0.14 |
| Cormia^[9]^ | 1934 | Warts | 12 | Yes | No | Phenol | SAS; 16.7% complete response |
| Total infectious studies: 9; total subjects: 838 | | | | | | | |
| Leong *et al*^[10]^ | 1999 | Melanoma | 35 | Yes | No | BCG, GM-CSF | SAS; ATV + GM-CSF had 20.0% CR or PR. No response in subjects given WATV + BCG |
| Ellem *et al*^[11]^ | 1997 | Melanoma | 1 | Yes | No | GM-CSF transfection | Case report; PR |
| Kusumoto *et al*^[12]^ | 2001 | Melanoma | 9 | Yes | No | GM-CSF transfection | SAS; 11.1% with PR; 88.9% no response noted |
| McCune *et al*^[13]^ | 1990 | Melanoma | 18 | No | No | *Cryptosporidium parvum*, allogenic material | SAS; DTH(+) patients survived for 69 months *vs.* DTH(−) patients survived for 44 months |
| Livingston *et al*^[14]^ | 1985 | Melanoma | 13 | Yes | No | Allogeneic material, VSV lysate | SAS; 7.7% developed antibodies to melanoma; no clinical responses recorded |
| Soiffer *et al*^[15]^ | 2003 | Melanoma | 34 | Yes | No | GM-CSF transfection | SAS; 29.4% 3-year survival rate |
| Chang *et al*^[16]^ | 2000 | melanoma | 5 | Yes | No | GM-CSF transfection | SAS; 20% had CR |
| Berd, Maguire, and Mastrangelo^[17]^ | 1986 | Melanoma | 19 | Yes | No | Cyclophosphamide | RCT; WATV alone elicited no regression, WATV + CY had 22.2% CR |
| Berd *et al*^[18]^ | 1990 | Melanoma | 40 | Yes | No | BCG | SAS; 10.0% had CR, 17.5% had PR |
| Total melanoma studies 9; total subjects: 174 | | | | | | | |
| Sobol *et al*^[19]^ | 1999 | CRC | 10 | Yes | No | Autologous fibroblast gene-modified to express IL2 | SAS; 30.0% had SD, which eventually progressed |
| Gianneschi *et al*^[20]^ | 2022 | CRC | 1 | Yes | No | Ground glass | Case report; CR for 2 years, then relapsed |
| Imaoka *et al*^[21]^ | 2017 | CRC, gallbladder | 2 | Yes | No | WATV alone | Case report; both had CR after 5 years |
| Ockert *et al*^[22]^ | 1996 | CRC | 57 | Yes | No | NDV, BCG | SAS; 97.9% ATV/NDV had 2-year survival, 66.7% of ATV/BCG had 2-year survival; mean survival of historical controls, 73.8% |
| Harris *et al*^[23]^ | 2000 | CRC | 205 | Yes | No | BCG | RCT; the greater the induration response to vaccination, the better the prognosis; 5-year survival and DFS for induration sizes < 5 mm, 5–10 mm, or >10 mm were 45.0%, 74.7%, and 84.6%; and 42.9%, 69.6%, and 79.3%, respectively. |
| Hoover *et al*^[24]^ | 1993 | CRC | 41 | Yes | No | BCG | RCT; median follow-up of 93 months, OS 83.3% in vaccinated, and 52.2% in the control (*P* = 0.02; HR, 3.97) DFS 66.7% vaccinated and 43.5% in the control (*P* = 0.04; HR, 2.67) |
| Vermorken *et al*^[25]^ | 1999 | CRC | 128 | Yes | Yes | BCG | RCT; recurrence for both stage II and III vaccinated 19.5% and control 31.7% *P* = 0.02. Stage II recurrence-free at 4 years was vaccinated = 88.0% and control = 74.0% (*P* = 0.01). In stage II, recurrence-free interval *P* = 0.01 and RFS *P* = 0.03 |
| Total CRC studies 7; total subjects: 444 | | | | | | | |
| Ohno^[26]^ | 2003 | HCC | 12 | Yes | No | Cytokine microparticle, tuberculin | SAS; 75.0% (all with DTH response) were RFS for 11–21 months; 1-year DFS in historical controls was between 35.9% and 37.5%, *P* < 0.05 |
| Huang *et al*^[27]^ | 1995 | HCC | 30 | UNR | No | Heat solidification | RCT; 2-year survival rate in the WATV arm was 60% compared to the control arm (13.5%) |
| Peng *et al*^[28]^ | 2006 | HCC | 32 | Yes | No | GM-CSF, hIL-2, tuberculin | RCT; the recurrence rate in the vaccine group (1 year, 12.6%; 2 years, 35.9%; 3 years, 54.0%); control group (1 year, 31.6%; 2 years, 61.3%; 3 years, 72.1%; *P* = 0.04) |
| Kuang *et al*^[29]^ | 2004 | HCC | 19 | Yes | No | hGM-CSF, hIL-2, tuberculin | RCT; median follow-up of 15 months, the risk of recurrence in vaccinated patients was reduced by 81.0% (95% confidence interval, 33.0–95.0%; *P* < 0.01). Vaccination significantly prolonged the time to first recurrence (*P* < 0.01) and improved RFS (*P* < 0.01) and OS rates (*P* = 0.01). |
| Total HCC studies: 4; total subjects: 93 | | | | | | | |
| Simons *et al*^[30]^ | 1997 | RCC | 18 | Yes | No | GM-CSF transfection | SAS; 5.6% had PR and regression of metastases for 7 months. No other patient responded. |
| May *et al*^[31]^ | 2010 | RCC | 692 | Yes | No | WATV alone* | RCT; patients with (more aggressive) pT3 stage RCC revealed 5- and 10-year survival rates of 71.3% and 53.6% in the study group and 65.4% and 36.2% in the control group (*P* = 0.02) |
| Galligioni *et al*^[32]^ | 1996 | RCC | 60 | Yes | No | BCG | RCT; 5-year DFS was 63% for the treatment arm and 72.0% for the control arm (not statistically significant); 5-year OS was 69.0% in the vaccine group and 78.0% in the control |
| Jocham *et al*^[33]^ | 2004 | RCC | 177 | Yes | No | WATV alone* | RCT; 5-year and 70-month PFS rates were 77.4% and 72%, respectively, in the vaccine group and 67.8% and 59.3%, respectively, in the control group |
| Doehn *et al*^[34]^ | 2007 | RCC | 233 | UNR | No | WATV alone* | RCT; PFS and OS were both statistically significant in favor of the Reniale group (*P* = 0.02, log-rank test, for PFS and *P* < 0.04, log-rank test, for OS) |
| Repmann, Wagner, and Richter^[35]^ | 1997 | RCC | 116 | Yes | No | BCG | SAS; RCC stage T3N0M0; 5-year OS was 77.5% in vaccinated patients *vs.* 25.0% in the historical control group; *P* < 0.01 log-rank test); 5-year PFS was 68.2% in the vaccine group *vs.* 19.4% in the historical control group, *P* < 0.0001 |
| Total RCC studies 6; total subjects: 1296 | | | | | | | |
| Cunningham *et al*^[36]^ | 1969 | Mixed study | 36 | Yes | Yes | Rabbit gamma globulin | SAS; 8.3% temporary remission, 5.5% SD, 86.1% PD |
| Czajkowski *et al*^[37]^ | 1967 | Mixed study | 14 | Yes | No | Rabbit gamma globulin | SAS; 14.3% had 4-year DFS, 14.3% 4-year SD |
| Finney, Byers, and Wilson^[38]^ | 1960 | Mixed study | 9 | Yes | No | Freund’s, penicillin, streptomycin | SAS; vaccine increased antibody titers compared to irradiation |
| Mahvi *et al*^[39]^ | 2002 | Mixed study | 17 | Yes | Yes | GM-CSF transfection | SAS; >4.5-year follow up 14/17 died, 1 PD, 1 DFS |
| Barve *et al*^[40]^ | 2022 | Mixed study | 13 | Yes | No | Vigil plasmid- transfected WATV + durvalumab | SAS; median PFS was 7.1 months |
| Total mixed studies 5; total subjects: 89 | | | | | | | |
| Holladay *et al*^41]^ | 1996 | CNS | 15 | Yes | No | BCG, IL2 | SAS; vaccination extended the time between surgery and recurrence, but all subjects had disease recurrence |
| Bota *et al*^[42,43]^ | 2018 | Glioblastoma | 5 | Yes | Yes | Allogenic material, GM-CSF, cyclophosphamide, bevacizumab | RCT; median OS of unblinded patients on vaccine at randomization or crossover is 328 days after the first study treatment, compared to 182 days for those randomized to placebo + bevacizumab who did not cross over. |
| Ishikawa *et al*^[44]^ | 2007 | Glioblastoma | 12 | Yes | No | BCG + tuberculin microparticles + soluble tuberculin | SAS; 8.3% CR, 8.3% PR, 16.7 minor response, 8.3% SD, 58.3% PD. 25% survived for ≥20 months after AFTV inoculation. |
| Sloan *et al*^[45]^ | 2000 | Malignant glioma | 19 | Yes | No | GM-CSF | SAS; 47.4% SD, 36.8% PR, 10.5% progressive disease, 5.2% CR |
| Total CNS cancer studies 4; total subjects: 51 | | | | | | | |
| Kuranishi and Ohno^46]^ | 2013 | Breast cancer | 1 | Yes | No | ATV alone | Case report; DFS over 4 years |
| Total breast cancer studies 1; total subjects: 1 | | | | | | | |
| Graham and Graham^[47]^ | 1969 | Pseudomyxoma peritonei | 5 | Yes | No | Freund’s, *Mycobacterium butyricum* or *Bordetella pertussis* | SAS; median OS = 3.15 years |
| Graham and Graham^[48]^ | 1959 | Gynecologic | 101 | UNR | No | Undisclosed, likely BCG | SAS; 54.5% lived for ≥7 months, 13.9% DFS after 30 months |
| Simons *et al*^[49]^ | 1999 | Prostate | 8 | Yes | No | GM-CSF transfection | SAS; 100% had DP |
| Crowther *et al*^[50]^ | 1978 | Ovarian | 17 | Yes | No | BCG | SAS; median survival 24 months in the vaccine group and 12 months in the historical control |
| Oh *et al*^[51]^ | 2016 | Ovarian | 31 | Yes | No | Vigil plasmid-transfected WATV | RCT; RFS from time of procurement was improved (mean 826 days/median 604 days in the Vigil arm from mean 481 days/median 377 days in the control arm, *P* = 0.03) |
| Rocconi *et al*^[52]^ | 2020 | Ovarian | 47 | Yes | No | Vigil plasmid-transfected WATV | RCT; RFS was 11.5 months (95% CI: 7.5–not reached) for the treatment arm *vs.* 8.4 months (95% CI: 7.9–15.5) for the placebo (HR: 0.69, 90% CI: 0.44–1.07; one-sided *P* = 0.08) |
| Rocconi *et al*^[53]^ | 2022 | Ovarian | 24 | Yes | Yes | Vigil plasmid-transfected WATV + atezolizumab | SAS; the exploratory subset analysis of BRCA suggested improved OS benefit (not reached in Vigil-1st *vs.* 5.2 months in Atezo-1st, HR 0.16, *P* = 0.03) |
| Total gynecologic cancer studies 7; total subjects: 233 | | | | | | | |
| Salgia *et al*^[54]^ | 2003 | NSCLC | 34 | Yes | No | GM-CSF transfection | SAS; 14.7% stable, 5.9% DFS, 2.9% mixed response |
| Nemunaitis *et al*^[55]^ | 2006 | NSCLC | 49 | Yes | Yes | Allogeneic GM-CSF-secreting cell | RCT; median PFS, median OS, and 1-year survival were 4.4 months, 7.0 months, and 31%, respectively, in the vaccinated group and 3.7 months, 5.4 months, and 22%, respectively, in the control group |
| Total lung cancer studies 2; total subjects: 83 | | | | | | | |
| Aswaq, Richards,and Mcfadden^[56]^ | 1964 | Carcinomas, unspecified | 21 | Yes | No | Freund’s | SAS; no difference in clinical course between ATV and historical control |
| Total carcinoma studies: 1; total subjects: 21 | | | | | | | |

*WATV was incubated with interferon-gamma and tocopherol-acetate to increase MHC expression. AFTV: Autologous formalin- fixed tumor vaccine; ATV: Autologous therapeutic vaccine; BCG: Bacillus Calmette-Guérin; BRCA: Breast cancer gene; CI: Confidence interval; CNS: Central nervous system; CR: Complete response; CRC: Colorectal cancer; CSF: Colony-stimulating factor; CY: Cyclophosphamide; DFS: Disease-free survival; DP: Disease progression; DTH: Delayed-type hypersensitivity; GM-CSF: Granulocyte-macrophage colony-stimulating factor; HCC: Hepatocellular carcinoma; hGM-CSF: Human granulocyte-macrophage colony-stimulating factor; hIL: Human interleukin; HR: Hazard ratio; IL: Interleukin; MHC: Major histocompatibility complex; NDV: New Castle disease virus; NSCLC: Non-small cell lung cancer; OS: Overall survival; PD: Progressive disease; PFS: Progression free survival; PR: Partial response; pT3: pathological primary tumor extending into major veins or perinephric tissue; RCC: Renal cell carcinoma; RCT: Randomized controlled trial; RFS: Recurrence-free survival; SAS: Single-arm study; SD: Stable disease; UNR: Unreported; VSV: vesicular stomatitis virus; WATV: Whole-tissue autologous therapeutic vaccine.

**References**

[1] Oleske JM, Kushnick T. Juvenile papilloma of the larynx. Am J Dis Child. 1971;121:417–419. <https://doi.org/10.1001/archpedi.1971.02100160087011>.

[2] Oleske J. Phase II study of immunotherapy with autogenous papilloma vaccine in patients with recurrent juvenile papilloma of the larynx. NCT00002454. Bethesda: National Institutes of Health, 1999. [Accessed on 2011 July 26].

[3] Shipkowitz NL, Holper JC, Worland MC, Holinger PH. Evaluation of an autogenous laryngeal papilloma vaccine. Laryngoscope 1967;77:1047–1066. doi: 10.1288/00005537-196706000-00011.

[4] Stephens CB, Arnold GE, Butchko GM, Hardy CL. Autogenous vaccine treatment of juvenile laryngeal papillomatosis. Laryngoscope 1979;89:1689–1696. doi: 10.1002/lary.5540891018.

[5] Powell LC Jr, Pollard M, Jinkins JL Sr. Treatment of condyloma acuminata by autogenous vaccine. South Med J 1970;63:202–205. doi: 10.1097/00007611-197002000-00017.

[6] Biberstein H. Immunization therapy of warts. Arch Derm Syphilol 1944;50:12–22. doi: 10.1001/archderm.1944.01510130015005.

[7] Abcarian H, Sharon N. Long-term effectiveness of the immunotherapy of anal condyloma acuminatum. Dis Colon Rectum 1982;25:648–651. doi: 10.1007/BF02629533.

[8] Malison MD, Morris R, Jones LW. Autogenous vaccine therapy for condyloma acuminatum. A double-blind controlled study. Br J Vener Dis 1982;58:62–65. doi: 10.1136/sti.58.1.62.

[9] Cormia FE. Autolysate therapy for verruca vulgaris. Arch Derm Syphilol 1934;30:44–48. doi: 10.1001/archderm.1934.01460130052009.

[10] Leong SPL, Enders-Zohr P, Zhou YM, Stuntebeck S, Habib FA, Allen RE Jr, et al. Recombinant human granulocyte macrophage-colony stimulating factor (rhGM-CSF) and autologous melanoma vaccine mediate tumor regression in patients with metastatic melanoma. J Immunother 1999;22:166–174. doi: 10.1097/00002371-199903000-00008.

[11] Ellem KA, O’Rourke MG, Johnson GR, Parry G, Misko IS, Schmidt CW, et al. A case report: Immune responses and clinical course of the first human use of granulocyte/macrophage-colony-stimulating-factor-transduced autologous melanoma cells for immunotherapy. Cancer Immunol Immunother 1997;44:10–20. doi: 10.1007/s002620050349.

[12] Kusumoto M, Umeda S, Ikubo A, Aoki Y, Tawfik O, Oben R, et al. Phase 1 clinical trial of irradiated autologous melanoma cells adenovirally transduced with human GM-CSF gene. Cancer Immunol Immunother 2001;50:373–381. doi: 10.1007/s002620100213.

[13] McCune CS, O’Donnell RW, Marquis DM, Sahasrabudhe DM. Renal cell carcinoma treated by vaccines for active specific immunotherapy: Correlation of survival with skin testing by autologous tumor cells. Cancer Immunol Immunother 1990;32:62–66. doi: 10.1007/BF01741726.

[14] Livingston PO, Albino AP, Chung TJ, Real FX, Houghton AN, Oettgen HF, et al. Serological response of melanoma patients to vaccines prepared from VSV lysates of autologous and allogeneic cultured melanoma cells. Cancer 1985;55:713–720. doi: 10.1002/1097-0142(19850215)55:4<713::aid-cncr2820550407>3.0.co;2-d.

[15] Soiffer R, Hodi FS, Haluska F, Jung K, Gillessen S, Singer S, et al. Vaccination with irradiated, autologous melanoma cells engineered to secrete granulocyte-macrophage colony-stimulating factor by adenoviral-mediated gene transfer augments antitumor immunity in patients with metastatic melanoma. J Clin Oncol 2003;21:3343–3350. doi: 10.1200/JCO.2003.07.005.

[16] Chang AE, Li Q, Bishop DK, Normolle DP, Redman BD, Nickoloff BJ. Immunogenetic therapy of human melanoma utilizing autologous tumor cells transduced to secrete granulocyte-macrophage colony-stimulating factor. Hum Gene Ther 2000;11:839–850. doi: 10.1089/10430340050015455.

[17] Berd D, Maguire HC Jr, Mastrangelo MJ. Induction of cell-mediated immunity to autologous melanoma cells and regression of metastases after treatment with a melanoma cell vaccine preceded by cyclophosphamide. Cancer Res 1986;46:2572–2577.

[18] Berd D, Maguire HC Jr, McCue P, Mastrangelo MJ. Treatment of metastatic melanoma with an autologous tumor-cell vaccine: Clinical and immunologic results in 64 patients. J Clin Oncol 1990;8:1858–1867. doi: 10.1200/JCO.1990.8.11.1858.

[19] Sobol RE, Shawler DL, Carson C, Van Beveren C, Mercola D, Fakhrai H, et al. Interleukin 2 gene therapy of colorectal carcinoma with autologous irradiated tumor cells and genetically engineered fibroblasts: A Phase I study. Clin Cancer Res 1999;5:2359–2365.

[20] Gianneschi G, Scolpino A, Dominguez J Jr, Oleske JM. Autologous therapeutic vaccine for stage IV metastatic colon cancer: A case report. Br J Med Health Sci 2022;4:1186–1188.

[21] Imaoka Y, Kuranishi F, Miyazaki T, Yasuda H, Ohno T. Long-lasting complete response status of advanced stage IV gall bladder cancer and colon cancer after combined treatment including autologous formalin-fixed tumor vaccine: Two case reports. World J Surg Oncol 2017;15:170. doi: 10.1186/s12957-017-1245-x.

[22] Ockert D, Schirrmacher V, Beck N, Stoelben E, Ahlert T, Flechtenmacher J, et al. Newcastle disease virus-infected intact autologous tumor cell vaccine for adjuvant active specific immunotherapy of resected colorectal carcinoma. Clin Cancer Res 1996;2:21–28.

[23] Harris JE, Ryan L, Hoover HC Jr, Stuart RK, Oken MM, Benson AB 3rd, et al. Adjuvant active specific immunotherapy for stage II and III colon cancer with an autologous tumor cell vaccine: Eastern Cooperative Oncology Group Study E5283. J Clin Oncol 2000;18:148–157. doi: 10.1200/JCO.2000.18.1.148.

[24] Hoover HC Jr, Brandhorst JS, Peters LC, Surdyke MG, Takeshita Y, Madariaga J, et al. Adjuvant active specific immunotherapy for human colorectal cancer: 6.5-year median follow-up of a phase III prospectively randomized trial. J Clin Oncol 1993;11:390–399. doi: 10.1200/JCO.1993.11.3.390.

[25] Vermorken JB, Claessen AM, van Tinteren H, Gall HE, Ezinga R, Meijer S, et al. Active specific immunotherapy for stage II and stage III human colon cancer: A randomised trial. Lancet 1999;353:345–350. doi: 10.1016/S0140-6736(98)07186-4.

[26] Ohno T. Autologous cancer vaccine: A novel formulation. Microbiol Immunol 2003;47:255–263. doi: 10.1111/j.1348-0421.2003.tb03393.x.

[27] Huang J, Zhang Z, Huang H, He Y. Study on solidified tumor vaccine prepared from autogenous cancerous tissue. Chin J Cancer Res 1995;7:225–229. doi: 10.1007/BF03023479.

[28] Peng B, Liang L, Chen Z, He Q, Kuang M, Zhou F, et al. Autologous tumor vaccine lowering postsurgical recurrent rate of hepatocellular carcinoma. Hepatogastroenterology 2006;53:409–414.

[29] Kuang M, Peng BG, Lu MD, Liang LJ, Huang JF, He Q, et al. Phase II randomized trial of autologous formalin-fixed tumor vaccine for postsurgical recurrence of hepatocellular carcinoma. Clin Cancer Res 2004;10:1574–1579. doi: 10.1158/1078-0432.ccr-03-0071.

[30] Simons JW, Jaffee EM, Weber CE, Levitsky HI, Nelson WG, Carducci MA, et al. Bioactivity of autologous irradiated renal cell carcinoma vaccines generated by ex vivo granulocyte-macrophage colony-stimulating factor gene transfer. Cancer Res 1997;57:1537–1546.

[31] May M, Brookman-May S, Hoschke B, Gilfrich C, Kendel F, Baxmann S, et al. Ten-year survival analysis for renal carcinoma patients treated with an autologous tumour lysate vaccine in an adjuvant setting. Cancer Immunol Immunother 2010;59:687–695. doi: 10.1007/s00262-009-0784-6.

[32] Galligioni E, Quaia M, Merlo A, Carbone A, Spada A, Favaro D, et al. Adjuvant immunotherapy treatment of renal carcinoma patients with autologous tumor cells and Bacillus Calmette-Guèrin: Five-year results of a prospective randomized study. Cancer 1996;77:2560–2566. doi: 10.1002/(SICI)1097-0142(19960615)77:12<2560::AID-CNCR20>3.0.CO;2-P.

[33] Jocham D, Richter A, Hoffmann L, Iwig K, Fahlenkamp D, Zakrzewski G, et al. Adjuvant autologous renal tumour cell vaccine and risk of tumour progression in patients with renal-cell carcinoma after radical nephrectomy: Phase III, randomised controlled trial. Lancet 2004;363:594–599. doi: 10.1016/S0140-6736(04)15590-6.

[34] Doehn C, Richter A, Theodor RA, Lehmacher W, Jocham D. 500: An adjuvant vaccination with Reniale prolongs survival in patients with renal cell carcinoma following radical nephrectomy: Secondary analysis of a multicenter phase-III trial. J Urol 2007;177:167–167. doi: 10.1016/S0022-5347(18)30740-7.

[35] Repmann R, Wagner S, Richter A. Adjuvant therapy of renal cell carcinoma with active-specific-immunotherapy (ASI) using autologous tumor vaccine. Anticancer Res 1997;17:2879–2882.

[36] Cunningham TJ, Olson KB, Laffin R, Horton J, Sullivan J. Treatment of advanced cancer with active immunization. Cancer 1969;24:932–937. doi: 10.1002/1097-0142(196911)24:5<932::aid-cncr2820240510>3.0.co;2-5.

[37] Czajkowski NP, Rosenblatt M, Wolf PL, Vazquez J. A new method of active immunisation to autologous human tumour tissue. Lancet 1967;290:905–909. doi: 10.1016/S0140-6736(67)90229-2.

[38] Finney JW, Byers EH, Wilson RH. Studies in tumor autoimmunity. Cancer Res 1960;20:351–356.

[39] Mahvi DM, Shi FS, Yang NS, Weber S, Hank J, Albertini M, et al. Immunization by particle-mediated transfer of the granulocyte-macrophage colony-stimulating factor gene into autologous tumor cells in melanoma or sarcoma patients: Report of a phase I/IB study. Hum Gene Ther 2002;13:1711–1721. doi: 10.1089/104303402760293556.

[40] Barve M, Aaron P, Manning L, Bognar E, Wallraven G, Horvath S, et al. Pilot study of combination Gemogenovatucel-T (Vigil) and durvalumab in women with relapsed BRCA-wt triple-negative breast or ovarian cancer. Clin Med Insights Oncol 2022;16:11795549221110501. doi: 10.1177/11795549221110501.

[41] Holladay FP, Heitz-Turner T, Bayer WL, Wood GW. Autologous tumor cell vaccination combined with adoptive cellular immunotherapy in patients with grade III/IV astrocytoma. J Neurooncol 1996;27:179–189. doi: 10.1007/BF00177482.

[42] Bota DA, Chung J, Dandekar M, Carrillo JA, Kong XT, Fu BD, et al. Phase II study of ERC1671 plus bevacizumab versus bevacizumab plus placebo in recurrent glioblastoma: Interim results and correlations with CD4+ T-lymphocyte counts. CNS Oncol 2018;7:CNS22. doi: 10.2217/cns-2018-0009.

[43] Bota D, Taylor T, Kong XT, Fu B, Hsu F, Strik A, et al. CTIM-09. Double-blinded, placebo controlled phase 2 study of ERC1671 in recurrent glioblastoma: vaccine overall survival in bevacizumab naive and bevacizumab resistant patients. Neuro Oncol 2020;22(Suppl 2):ii34. doi: 10.1093/neuonc/noaa215.143.

[44] Ishikawa E, Tsuboi K, Yamamoto T, Muroi A, Takano S, Enomoto T, et al. Clinical trial of autologous formalin-fixed tumor vaccine for glioblastoma multiforme patients. Cancer Sci 2007;98:1226–1233. doi: 10.1111/j.1349-7006.2007.00518.x.

[45] Sloan AE, Dansey R, Zamorano L, Barger G, Hamm C, Diaz F, et al. Adoptive immunotherapy in patients with recurrent malignant glioma: Preliminary results of using autologous whole-tumor vaccine plus granulocyte-macrophage colony-stimulating factor and adoptive transfer of anti-CD3-activated lymphocytes. Neurosurg Focus 2000;9:e9. doi: 10.3171/foc.2000.9.6.10.

[46] Kuranishi F, Ohno T. Eradication of breast cancer with bone metastasis by autologous formalin-fixed tumor vaccine (AFTV) combined with palliative radiation therapy and adjuvant chemotherapy: A case report. World J Surg Oncol 2013;11:127. doi: 10.1186/1477-7819-11-127.

[47] Graham JB, Graham R. Pseudomyxoma peritonei treated with autogenous vaccine. Clin Obstet Gynecol 1969;12:955–957. doi: 10.1097/00003081-196912040-00008.

[48] Graham JB, Graham RM. The effect of vaccine on cancer patients. Surg Gynecol Obstet 1959;109:131–138. doi: 10.1097/00006534-195911000-00016.

[49] Simons JW, Mikhak B, Chang JF, DeMarzo AM, Carducci MA, Lim M, et al. Induction of immunity to prostate cancer antigens: Results of a clinical trial of vaccination with irradiated autologous prostate tumor cells engineered to secrete granulocyte-macrophage colony-stimulating factor using ex vivo gene transfer. Cancer Res 1999;59:5160–5168.

[50] Crowther ME, Levin L, Poulton TA, Saffrey MJ, Curling OM, Hudson CN. Active specific immunotherapy in ovarian cancer. Recent Results Cancer Res 1978;68:166–173. doi: 10.1007/978-3-642-81332-0_25.

[51] Oh J, Barve M, Matthews CM, Koon EC, Heffernan TP, Fine B, et al. Phase II study of Vigil® DNA engineered immunotherapy as maintenance in advanced stage ovarian cancer. Gynecol Oncol 2016;143:504–510. doi: 10.1016/j.ygyno.2016.09.018.

[52] Rocconi RP, Grosen EA, Ghamande SA, Chan JK, Barve MA, Oh J, et al. Gemogenovatucel-T (Vigil) immunotherapy as maintenance in frontline stage III/IV ovarian cancer (VITAL): A randomised, double-blind, placebo-controlled, phase 2b trial. Lancet Oncol 2020;21:1661–1672. doi: 10.1016/S1470-2045(20)30533-7.

[53] Rocconi RP, Stevens EE, Bottsford-Miller JN, Ghamande SA, Elder J, DeMars LL, et al. Proof of principle study of sequential combination atezolizumab and Vigil in relapsed ovarian cancer. Cancer Gene Ther 2022;29:369–382. doi: 10.1038/s41417-021-00317-5.

[54] Salgia R, Lynch T, Skarin A, Lucca J, Lynch C, Jung K, et al. Vaccination with irradiated autologous tumor cells engineered to secrete granulocyte-macrophage colony-stimulating factor augments antitumor immunity in some patients with metastatic non–small-cell lung carcinoma. J Clin Oncol 2003;21:624–630. doi: 10.1200/JCO.2003.03.091.

[55] Nemunaitis J, Jahan T, Ross H, Sterman D, Richards D, Fox B, et al. Phase 1/2 trial of autologous tumor mixed with an allogeneic GVAX® vaccine in advanced-stage non-small-cell lung cancer. Cancer Gene Ther 2006;13:555–562. doi: 10.1038/sj.cgt.7700922.

[56] Aswaq M, Richards V, Mcfadden S. Immunologic response to autologous cancer vaccine. Arch Surg 1964;89:485–487. doi: 10.1001/archsurg.1964.01320030075012.
